# Pathogen and Patient Characteristics and the Severity of Viral Respiratory Infections in Children

**DOI:** 10.1001/jamanetworkopen.2026.0129

**Published:** 2026-02-26

**Authors:** Cristina Moracas, Marco Poeta, Elisabetta Venturini, Amelia Licari, Valeria Garbo, Marta Stracuzzi, Ester Del Tufo, Laura Petrarca, Marco Maglione, Claudio Cafagno, Agnese Tamborino, Sofia Sgubbi, Andrea Lo Vecchio, Claudia Colomba, Vania Giacomet, Luisa Galli, Gian Luigi Marseglia, Alfredo Guarino

**Affiliations:** 1 Pediatric Infectious Disease Unit, Department of Maternal and Child Health, University Hospital Federico II, Naples, Italy; 2 Department of Public Health, Experimental and Forensic Medicine, University of Pavia, Pavia, Italy; 3 Department of Translational Medical Science, University of Naples Federico II, Naples, Italy; 4 Infectious Diseases Unit, Meyer Children’s Hospital, Istituto di Ricovero e Cura a Carattere Scientifico (IRCCS), Florence, Italy; 5 Pediatric Unit, Department of Clinical, Surgical, Diagnostic and Pediatric Sciences, University of Pavia, Pavia, Italy; 6 Pediatric Clinic, Fondazione IRCCS Policlinico, San Matteo, Pavia, Italy; 7 Department of Health Promotion, Maternal and Infant Care, Internal Medicine and Medical Specialties, University of Palermo, Palermo, Italy; 8 Pediatric Infectious Disease Unit, Department of Pediatrics, Azienda Socio Sanitaria Territoriale Fatebenefratelli L. Sacco, University of Milan, Milan, Italy; 9 Department of Pediatrics, S. Maria delle Grazie Pozzuoli Hospital, Naples, Italy; 10 Department of Maternal, Infantile and Urological Sciences, Sapienza University of Rome, Rome, Italy; 11 Pediatric Emergency Department, Santobono-Pausilipon Children’s Hospital, Naples, Italy; 12 Division of Pediatric Infectious Diseases, Children’s Hospital Giovanni XXIII, Bari, Italy; 13 Division of Pediatric Infectious Diseases, G. Di Cristina Hospital, Azienda ospedaliera di Rilievo Nazionale e di Alta Specializzazione Civico Di Cristina Benfratelli, Palermo, Italy; 14 Department of Health Sciences, University of Florence, Florence, Italy

## Abstract

**Importance:**

Respiratory infections are a leading cause of pediatric hospitalization and impose a significant burden on health care systems.

**Objectives:**

To evaluate clinical outcomes and risk factors for severe disease in hospitalized children with viral respiratory infections, focusing on the role of etiologies, comorbidities, age, and coinfections.

**Design, Setting, and Participants:**

This multicenter observational cohort study is part of an active pediatric surveillance system established in September 2023, within the framework of the European Union–funded National Recovery and Resilience Plan. The One Health Basic and Translational Actions Addressing Unmet Needs on Emerging Infectious Diseases (INF-ACT) network, which includes 12 pediatric infectious disease referral centers across Italy, is designed to enable early detection of emerging infectious threats in children. The network is based on 3 pillars: monthly online meetings, a register of cases of unusual severity or induced by unusual pathogens, and a sample repository to investigate microbiology or host-related risk factors. Hospitalized children with respiratory symptoms and confirmed or suspected viral infection between September 2023 and December 2024 were eligible.

**Exposures:**

Type of respiratory virus, viral or bacterial coinfections, and presence of chronic comorbidities.

**Main Outcomes and Measures:**

Severity was assessed using a clinical severity score (CSS), with a score greater than 3 indicating severe disease. The CSS considered mechanical ventilator support, hospital admission, length of hospital stay, oxygen saturation level, and use of supplemental oxygen. Length of hospital stay, intensive care unit admission, and the need for oxygen therapy were also considered as individual outcomes.

**Results:**

Of 516 children hospitalized with respiratory symptoms (median [IQR] age, 13.3 [4.0-43.0] months; 288 male [55.8%]), 34 (6.6%) had severe disease (CSS >3). In multivariable analysis, among patients with respiratory symptoms, infection with respiratory syncytial virus (10 of 67 [14.9%]; adjusted odds ratio [AOR], 4.26 [95% CI, 1.80-10.10]) or influenza (14 of 104 [13.5%]; AOR, 4.13 [95% CI, 1.88-9.04]) and the presence of cardiac or pulmonary diseases (4 of 18 [22.2%]; AOR, 5.25 [95% CI, 1.47-18.78]) or congenital malformations (3 of 14 [21.4%]; AOR, 4.94 [95% CI, 1.23-19.93]) were associated with increased risk of severe infection. A higher ICU admission rate was observed in children with influenza infection (12 of 104 [11.5%] vs 9 of 412 [2.2%]; *P* < .001) and preexisting comorbidities (9 of 69 [13.0%] vs 12 of 447 [2.7%]; *P* < .001). Age and coinfections were not significantly associated with worse outcomes.

**Conclusions and Relevance:**

In this cohort study of hospitalized children, respiratory syncytial virus and influenza infections, as well as the presence of chronic comorbidities, were significantly associated with severe disease. These findings support the need for targeted prevention and vaccination strategies, as well as early identification of children at risk for severe disease. The INF-ACT network offered a scalable model for real-time infectious disease monitoring in pediatric populations.

## Key Points

**Question:**

How are pathogens, age, comorbidities, and coinfections associated with the severity of viral respiratory infections in children?

**Findings:**

In this cohort study of 516 children, respiratory syncytial virus infections, influenza infections, and the presence of preexisting chronic conditions were associated with significantly worse clinical outcomes, including clinical severity score, length of hospital stay, intensive care unit admission, and the need for oxygen therapy.

**Meaning:**

These results underscore the importance of targeted surveillance and preventive measures, including vaccination and early intervention, particularly for vulnerable pediatric subgroups.

## Teaser

This cohort study examines the association of risk factors and clinical outcomes with several viral respiratory infections among hospitalized children in a multicenter pediatric health care system in Italy.

## Introduction

Severe infections are among the most common causes of unplanned hospital admissions,^1^ with a high incidence of respiratory illnesses occurring in children, resulting in a substantial impact on health care resources.^2^ The COVID-19 pandemic had a massive impact on the global health system and highlighted a failure in preparedness for outbreaks, requiring a profound shift in the approach to infectious diseases.^3,4^ Public health measures such as masking, social distancing, and travel restrictions reduced children’s exposure to common pathogens, which hindered natural immunization and increased their susceptibility to other infections. This changed the epidemiology of viral respiratory infections in children and increased the severity of respiratory viral infections due to common pathogens^3,5,6^

Viral infections may present with a broad range of clinical symptoms and severity, ranging from mild, self-limiting symptoms, such as fever, upper respiratory symptoms, and gastrointestinal disorders, to more severe presentations, including respiratory failure, dehydration, and severe life-threatening complications.^7,8,9^ However, substantial interindividual variability in infection outcomes is observed even within populations infected by the same viral strain, reflecting a complex interplay between microorganism and host-specific factors.^10^

The COVID-19 pandemic had a massive impact on the global health system and highlighted a failure in preparedness for outbreaks, requiring a profound shift in the approach to infectious diseases.^4,5^ Different conditions, including concomitant infections, age at presentation, and preexisting comorbidities, have been considered to impact the clinical course of pediatric infections, although the relationship is not well established and the results of previous studies are conflicting.^11,12,13,14,15,16,17,18^

In this context, research efforts should be directed toward increasing the preparedness, readiness, and early response capacity of health systems to address emerging infectious disease threats. Identifying risk factors associated with the development of severe respiratory infections in pediatric patients, as well as the patient groups at greatest risk of severe disease, is essential to guide clinical practice and improve outcomes, thereby optimizing clinical management and allocating health care resources to specific needs.

In late 2023, we developed a clinical multicentric monitoring system for early detection of current and future potential infectious threats in Italy. This system detects both new emerging viruses and reemerging known pathogens with new clinical, biochemical, or epidemiological presentation, focusing on the pediatric setting. In the present work, we aimed to investigate factors that may confer a high susceptibility to severe infections in children, specifically to define the role of specific viral pathogens, comorbidities, age, and coinfections in shaping the clinical course of pediatric respiratory illness.

## Methods

### Study Design and Data Source

The cohort study was conducted within the framework of an active pediatric surveillance system established in September 2023 as part of a European Union (EU)–funded National Recovery and Resilience Plan (NRRP) project, known as One Health Basic and Translational Actions Addressing Unmet Needs on Emerging Infectious Diseases (INF-ACT). Currently, the surveillance system includes 12 Italian reference centers for pediatric infectious diseases, selected among university hospitals and large pediatric hospitals with a high level of specialization in pediatric infections. This initiative aims to identify infectious threats in Italy, focusing on the pediatric population and emerging and reemerging infections, and to assess protective and risk factors for infections in the post–COVID-19 era. This report was prepared in accordance with the Strengthening the Reporting of Observational Studies in Epidemiology (STROBE) reporting guideline for cohort studies. To minimize the significant clinical and immunological differences between pediatric and adult populations, the present study focused exclusively on children (patients aged <18 years).

### Ethical Issues

The study was conducted in accordance with the Declaration of Helsinki and was approved by the Ethics Committee for Biomedical Activities at the University of Naples Federico II. Written informed consent to use clinical data and biological samples was obtained from the parents of the children involved in the study.

### Network Components and Study Population

The network consisted of 3 arms. The first arm was an anonymized, password-protected database accessible only to study researchers to record features of pediatric patients. Inclusion criteria were age younger than 18 years; hospitalization from September 2023 in 1 of the 12 participating centers; and a confirmed or suspected viral infection presented with (1) acute gastroenteritis, (2) acute respiratory symptoms, (3) neurological symptoms, (4) fever lasting more than 4 days, (5) atypical skin manifestations, or (6) persistence of symptoms and undefined diagnosis after 7 days of hospitalization, atypical or severe presentation, or emerging viruses.

The second arm of the network consisted of monthly online meetings among study researchers, which provide an opportunity to exchange clinical information. During these meetings, researchers report severe, unexplained, or novel clinical manifestations observed during routine clinical activity, such as abnormal increases in the number of cases of a specific infection, diseases with unusual duration, atypical clinical features, unexpected mortality, or abnormal laboratory test results, with the aim of early identification of potential infectious threats. In 2023, the network was able to promptly detect the abnormal resurgence of pertussis cases,^19^ as well as an unusually high number of myocarditis cases related to parvovirus B19 infection.^20^

The third arm was the biological bank, a storage of biological samples collected in cases of severe disease, peculiar clinical presentation, or an unknown etiology, to have available biological samples for subsequent evaluations. For this study, only patients hospitalized from September 2023 to December 2024 for respiratory symptoms were selected.

### Definitions

Comorbidities included preexisting and chronic health conditions, defined as any medical condition that can be reasonably expected to last at least 12 months (unless death occurs) and to involve either several different organ systems or 1 organ system severely enough to require specialty pediatric care and hospitalization in a tertiary care center.^21,22^ Comorbidities were identified using diagnostic codes from the *International Classification of Diseases, 11th Revision*, according to the World Health Organization classification,^23^ and were further categorized into 5 groups: (1) endocrine and inherited metabolic diseases, including hormone glands disfunctions and deficit of enzymes or their cofactors that cause errors in metabolic processes; (2) immune or autoimmune diseases and immunocompromised conditions (transplant; ongoing chemotherapy or radiation therapy; immunomodulatory or biological drugs, or high-dose steroids given for less than 15 days before hospital admission; or human immunodeficiency virus infection); (3) cardiac or pulmonary diseases, including congenital heart diseases, ventricular disfunctions, lung malformations, and asthma; (4) neurological conditions, including epilepsy and cerebral palsy; and (5) congenital malformations, including chromosomal disorders and gastrointestinal or nephrological malformations.

All patients underwent nasal swab testing for detecting the following respiratory pathogens by real-time polymerase chain reaction (PCR): influenza virus, parainfluenza virus (PIV), human rhinovirus or enterovirus (HRV/ERV), human adenovirus (HAdV), human coronavirus (HCoV, including SARS-CoV-2 and others), respiratory syncytial virus (RSV), human metapneumovirus (hMPV) and human bocavirus (HBoV), *Bordetella pertussis* and *parapertussis*, *Chlamydophila pneumoniae,* and *Mycoplasma pneumoniae*.

Coinfection was defined as the simultaneous detection of 2 or more pathogens. In our analysis, we separately analyzed coinfections with respiratory viruses (including all viruses commonly affecting the respiratory system mentioned above), nonrespiratory viruses (viruses not primarily targeting the respiratory tract, including Epstein-Barr virus, cytomegalovirus, enterovirus, coxsackievirus, and herpes virus), and bacteria.

### Outcome Measures

To identify risk and protective factors of infection severity, a clinical severity score (CSS)^24^ was calculated for each patient, ranging from 0 to 6. Two points were assigned if the patient required mechanical ventilator support during the hospitalization, and 1 point was assigned for each of the following factors: hospital admission (applied to all children), hospitalization for 5 or more days, oxygen saturation less than 87%, or use of supplemental oxygen. Severe respiratory disease was defined as a CSS greater than 3.^25^

Length of hospital stay, intensive care unit (ICU) admission, and the need for oxygen therapy were also considered as individual outcomes, independent of the CSS context.

The following additional outcomes were considered as severity parameters for different categories of patients: death, need for intravenous fluids and steroid therapy, and clinical respiratory score (CRS), which includes several predictors of respiratory distress, including child clinical appearance, respiratory rate, thorax auscultation, use of accessory muscles, mental status, and oxygen saturation. Based on the total CRS, there can be 3 categories of respiratory distress: mild (**≤**3), moderate (4-7), and severe (8-12).^26^ All data refer to the most severe phase of the child’s clinical history.

### Statistical Analysis

Statistical analyses were performed with SPSS Statistics for Windows, version 29.0 (IBM Corp). Continuous variables were reported as means and SDs or medians and IQRs, according to their distribution, and compared using a *t* test or Mann-Whitney test, as appropriate. Categorical variables expressed as frequencies and percentages were compared using the Fisher exact test or χ^2^. Univariable logistic regression was used to investigate variables associated with infection severity, with risk expressed as odds ratios (ORs) with 95% CIs. The univariate analysis was exploratory, and no adjustment for multiple testing was applied. Multivariate models were used to account for simultaneous effects of covariates, expressed as adjusted ORs (AORs) with 95% CIs. A hierarchical backward model selection was performed, including all variables found to be significant in the univariate analysis. Models were compared using the Akaike information criterion (AIC): at each step, 1 covariate was removed, and covariates whose removal resulted in an increase in AIC were retained in the multivariate logistic regression model. To evaluate whether the association between selected risk factors and clinical severity varied by age, interaction terms were constructed by multiplying the main variable by centered age. Age was centered by subtracting the sample mean age from each individual age value (centered age = individual age − mean age). These interaction terms were included in the multivariable logistic regression models and tested for statistical significance. The level of significance was set at .05 (2-tailed).

## Results

A total of 668 hospitalized children with a confirmed or suspected viral infection who met the inclusion criteria were initially included in the database. Among them, 516 children (77.2%) with respiratory symptoms were included in the analysis (median [IQR] age, 13.3 [4.0-43.0] months; 288 male [55.8%] and 228 female [44.2%]) (Table 1), while 152 children (22.7%) with different clinical presentations (gastrointestinal, cutaneous, and neurological) in absence of a respiratory involvement were excluded. Children aged 0 to 60 months represented the highest proportion of hospital admissions (432 [83.7%]). Of all children included, 69 (13.4%) had a preexisting comorbidity. The prevalence of chronic conditions increased with age (*P* < .001). Data on prematurity (defined as birth before completion of 37 weeks’ gestation) were evaluated only for children younger than 1 year of age, and 3 infants (1.8%) were premature. The CSS showed a severe infection in 34 patients (6.6%). Influenza vaccination status was available for 307 patients, of whom only 23 (7.5%) had received the vaccine.

**Table 1.** Demographic Features, Viral Etiologies, and Outcomes of the Study Population

Characteristic	No. (%) (N = 516)
**Demographic features**	
Sex	
Male	288 (55.8)
Female	228 (44.2)
Age, mo	
Median (IQR)	13.3 (4.0-43.0)
0-3	119 (23.1)
4-12	136 (26.4)
13-60	177 (34.3)
>60	84 (16.3)
≥1 Comorbidity	69 (13.4)
Endocrine or inherited metabolic disease	10 (1.9)
Immune disease or immunocompromised	13 (2.5)
Cardiac or pulmonary diseases	18 (3.5)
Neurological conditions	14 (2.7)
Congenital malformations	14 (2.7)
**Outcomes**	
Clinical severity score >3	34 (6.6)
Oxygen therapy	148 (28.7)
Parenteral rehydration	216 (41.9)
Steroid therapy	203 (39.3)
Length of hospital stay, median (IQR), d	4 (3-7)
Intensive care unit admission	21 (4.1)
Death	3 (0.6)
**Etiologies**	
Rhinovirus/enterovirus	166 (32.2)
Adenovirus	134 (26.0)
Influenza virus	104 (20.2)
Coronavirus	72 (14.0)
Respiratory syncytial virus	67 (13.0)
Metapneumovirus	48 (9.3)
Parainfluenza virus	25 (4.8)
Bocavirus	24 (4.7)
No respiratory viruses detected	24 (4.7)
**Coinfections**	
Single respiratory virus infection	348 (67.4)
≥1 Viral coinfection	193 (37.4)
≥1 Viral respiratory coinfection	144 (27.9)
2 Respiratory viruses detected	139 (26.9)
3 Respiratory viruses detected	5 (1.0)
Nonrespiratory viral coinfection	51 (9.9)
Bacterial coinfection	148 (28.7)

### Potential Risk Factors for Severe Infection According to Etiology

Of the 516 respiratory samples analyzed from the corresponding patients, 492 (95.3%) were positive for at least 1 respiratory virus, whereas in 24 patients (4.7%), no respiratory virus was detected. Viral mono-infection was found in 348 samples (67.4%), and coinfection was observed in 144 specimens (27.9%).

Patients with influenza infection, compared with other infections, had a significantly higher ICU admission rate (12 of 104 [11.5%] vs 9 of 412 [2.2%]; *P* < .001) and length of hospital stay (mean [SD], 7.7 [8.9] days vs 5.5 [4.2] days; *P* = .02). RSV, HBoV, and hMPV had the highest rates of oxygen supplementation requirement (42 of 67 [62.7%], 12 of 24 [50.0%] and 21 of 48 [43.8%], respectively). Influenza, RSV, and HBoV were associated with a significantly higher CSS compared with all other cases, whereas the lowest mean value was observed for HCoV-infected children (eFigure 1 in Supplement 1).

**Figure 1.**
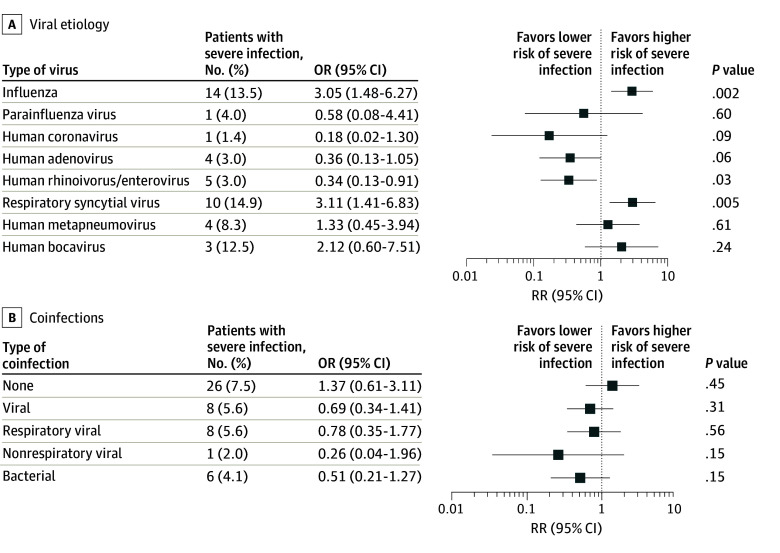
Forest Plots Showing Odds Ratios (ORs) for Severe Respiratory Infections by Viral Etiologies and Coinfections The number of patients with severe respiratory infection is defined as those with a clinical severity score greater than 3. Forest plots show odds ratios for severe infection by viral etiologies (A) and by coinfections, subdivided into viral respiratory and nonrespiratory coinfections, and bacterial coinfections (B).

Influenza and RSV infection were associated with increased odds of severe infection compared with other viruses (influenza: OR, 3.05 [95% CI, 1.48-6.27]; *P* = .002; RSV: OR, 3.11 [95% CI, 1.41-6.83]; *P* = .005). Conversely, HRV/ERV was associated with lower odds of severe disease (OR, 0.34 [95% CI, 0.13-0.91]; *P* = .03) (Figure 1A). Within the entire cohort, 3 deaths were observed, all in patients with influenza, due to a hyperinflammatory-related syndrome.

### Potential Risk Factors for Severe Infection According to Coinfections

Viral coinfections mostly involved 2 viruses (139 [26.9%]), whereas the detection of 3 viruses was less frequent (5 [1.0%]). The most frequent coinfecting pathogen was HAdV, detected in association with at least 1 other respiratory virus in 81 cases (60.4%). Coinfections with nonrespiratory viruses were detected in 51 of 516 patients (9.9%), and bacterial coinfections in 148 of 516 children (28.7%) (Table 1). Individuals with multiple viral detections did not experience worse outcomes and had a similar mean CSS (mean [SD] score, 1.8 [1.0] vs 1.8 [1.1]; *P* = .93) and length of hospital stay (mean [SD], 6.1 [4.8] days vs 6.8 [5.2] days; *P* = .12) (eFigure 2 in Supplement 1).

No significant association with increased risk of severe disease was observed for viral, nonrespiratory virus, or bacterial coinfections compared with patients with single respiratory viral infections (Figure 1B). Even after excluding HRV/ERV coinfections, due to uncertainty regarding their pathogenicity, no association between coinfection and disease severity was observed. Those findings were confirmed even when the analysis was stratified by pathogen groups.

Analyzing the distribution of coinfections across different age groups, we found that the rate of respiratory coinfections was higher in infants aged 3 to 12 months (43 of 136 [31.6%]) and in children aged 12 to 60 months (63 of 177 [35.6%]) compared with infants aged 0 to 3 months (19 of 119 [16.0%]) and older than 60 months (19 of 84 [22.6%]) (*P* < .001). In contrast, bacterial coinfections were more commonly observed in younger children. However, no significant interaction between centered age and respiratory coinfections was observed for disease severity.

### Potential Risk Factors for Severe Infection According to Age

**Figure 2.**
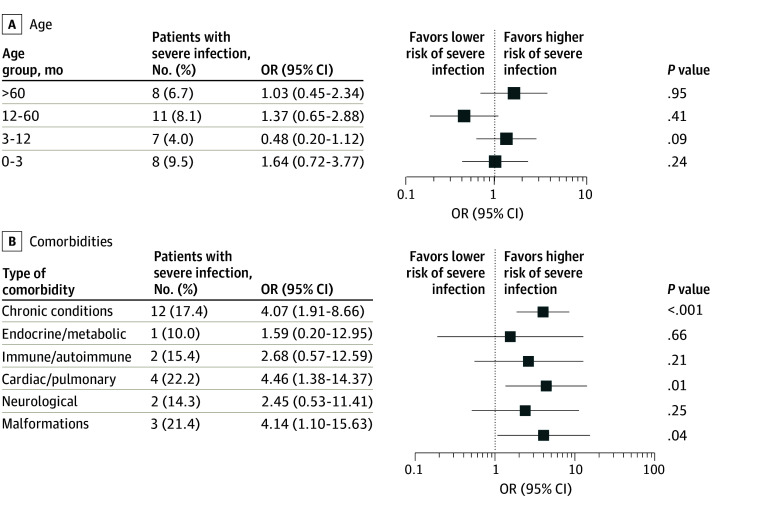
Forest Plots Showing Odds Ratios (ORs) for Severe Respiratory Infections by Age and Comorbidities The number of patients with severe respiratory infection is defined as those with a clinical severity score greater than 3. Forest plots show the odds ratios for severe infection by age (A) and by comorbidities, further subdivided into the following categories: endocrine and inherited metabolic diseases, immune or autoimmune diseases, cardiac or pulmonary diseases, neurological conditions, and congenital malformations (B).

ICU admission was more frequent in children older than 60 months (7 of 84 [8.3%] vs 14 of 432 [3.2%]; *P* = .04), whereas infants younger than 3 months received oxygen more frequently (42 of 119 [35.3%] vs 106 of 397 [26.7%]; *P* = .05) (eFigure 3A in Supplement 1). No differences were observed between age groups in CSS mean values, length of hospital stay, or mortality rate (eFigure 3B in Supplement 1). Age was not associated with an increased risk of severe clinical course of infection by univariate analysis (Figure 2A).

### Potential Risk Factors for Severe Infection According to Preexisting Comorbidities

Among the 69 children with comorbidities, 9 (13.0%) required ICU admission, compared with 12 of 447 children (2.7%) without underlying comorbidities (eFigure 4A in Supplement 1), resulting in increased odds (OR, 5.40 [95% CI, 2.20-13.45]; *P* < .001) of ICU admission in children with preexisting comorbidities. Among them, patients with congenital malformations had the highest rate of intensive care needs (3 of 14 [21.4%]). Patients with chronic conditions also experienced a longer duration of hospitalization (mean [SD], 7.8 [7.5] days vs 5.7 [5.2] days; *P* = .03) and a higher mean CSS (mean [SD] score, 2.3 [1.5] vs 1.7 [0.9]; *P* = .004) (eFigure 4B in Supplement 1).

In the univariate analysis, chronic conditions were associated with increased odds (OR, 4.07 [95% CI, 1.91-8.66]; *P* < .001) of developing a severe course of respiratory infection compared with children without chronic diseases. Among them, patients with cardiac or pulmonary diseases, as well as those with congenital malformations, were at the highest risk (Figure 2B). No significant interaction between centered age and comorbidities was observed for disease severity.

### Multivariate Analysis of Potential Risk Factors for Severe Infection 

**Figure 3.**
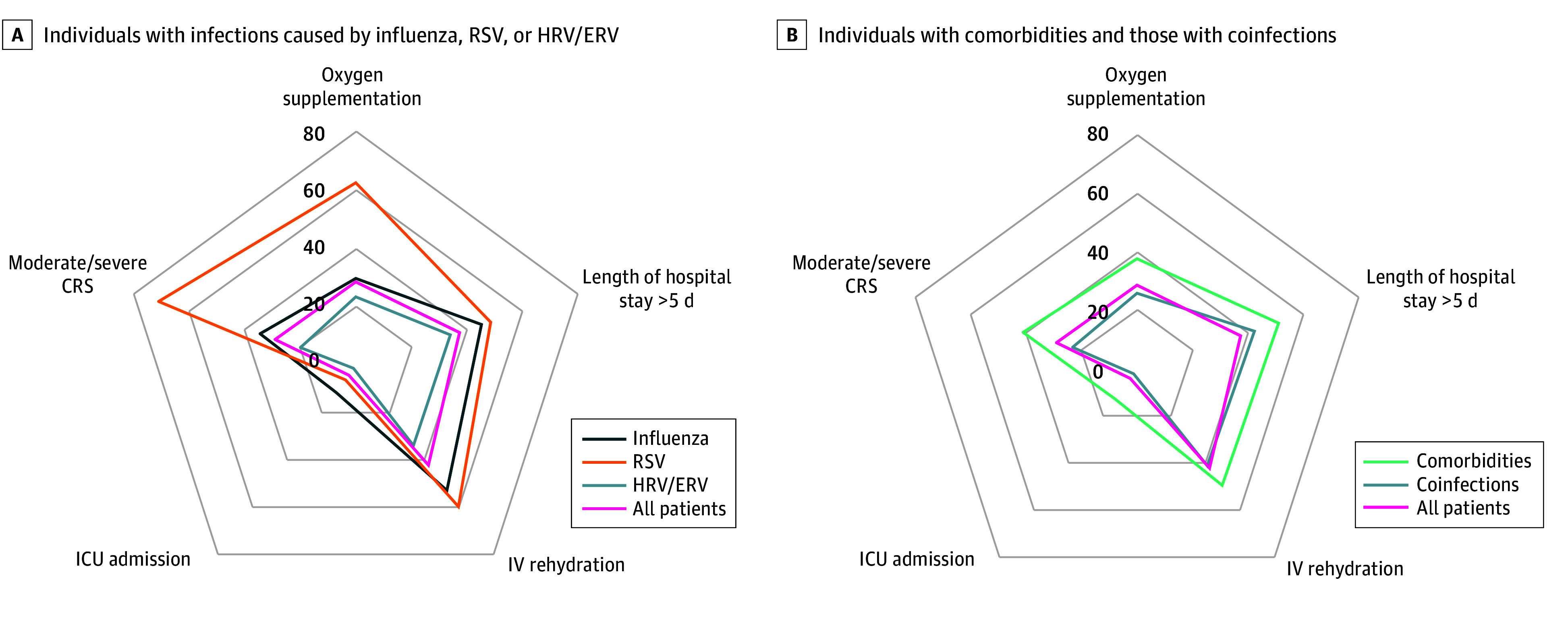
Radar Plots Comparing the Clinical Burden Associated With Different Infection Types, Coinfections, and Comorbidities Each axis corresponds to a clinical outcome (oxygen supplementation, prolonged hospitalization, IV rehydration, ICU admission, CRS severity), while polygons represent distinct patient groups: A, individuals with infections caused by influenza, RSV, or HRV/ERV; B, individuals with comorbidities or coinfections. CRS, clinical respiratory score; HRV/ERV, human rhinovirus/enterovirus; ICU indicates intensive care unit; IV, intravenous; RSV, respiratory syncytial virus.

In Figure 3, we provide a summary of the key clinical outcomes across patients with different infection types, coinfections, and comorbidities, graphically indicating the highest impact of influenza, RSV, and comorbidities. We built a multivariable model to identify parameters associated with clinical outcomes, including all variables found to be significant in the univariate analysis except HRV/ERV infection, which was excluded based on hierarchical model selection using the AIC. The model confirmed the results of the univariate analysis and showed that RSV infections were associated with worse clinical outcomes (AOR, 4.26 [95% CI, 1.80-10.10]; *P* < .001), as well as influenza infection (AOR, 4.13 [95% CI, 1.88-9.04]; *P* < .001) and the presence of preexistent chronic conditions, particularly cardiac or pulmonary diseases (AOR, 5.25 [95% CI, 1.47-18.78]; *P* = .01) and congenital malformations (AOR, 4.94 [95% CI, 1.23-19.93]; *P* = .03) (Table 2).

**Table 2.** Multivariate Logistic Regression Analysis of Severe Respiratory Infection by Viral Etiologies and Comorbidities^a^

Variable	Multivariable analysis	
	AOR (95% CI)	*P* value
Etiologies^b^		
Influenza virus	4.13 (1.88-9.04)	<.001
Respiratory syncytial virus	4.26 (1.80-10.10)	<.001
Comorbidities^b^		
Cardiac or pulmonary diseases	5.25 (1.47-18.78)	.01
Congenital malformations	4.94 (1.23-19.93)	.03

Abbreviation: AOR, adjusted odds ratio.

^a^ Patients with severe respiratory infection are defined as those with a clinical severity score greater than 3.

^b^ Includes selected variables with significant associations in univariate analysis.

## Discussion

This cohort study using a multicenter pediatric surveillance model, developed in collaboration with reference centers of pediatric infectious diseases in Italy, provides important insights into the factors associated with the severity of respiratory viral infections in hospitalized children. Our findings highlight the significant role of specific viral pathogens and preexisting comorbidities, rather than age and coinfections, in shaping the clinical course of respiratory infections in the pediatric population.

Previous studies investigating this issue were typically focused on a single viral etiology.^11,12,27,28^ To our knowledge, no prior research has comprehensively examined the clinical course of respiratory symptoms regardless of the identified pathogen; explored how coinfections, age, or preexisting comorbidities interact with each other in determining disease severity; or evaluated the comparative course of different viral infections due to different pathogens. With the cooperation of several centers distributed throughout Italy and a unified surveillance system that allowed for information sharing and discussion, a substantial amount of data collected during a single season was standardized and analyzed to identify key findings.

Our results demonstrate that infections caused by RSV and influenza, as mono-infections or as part of a coinfection, are strongly associated with more severe clinical outcomes. Conversely, infections with HRV/ERV were associated with a lower risk of severe illness, underscoring the heterogeneity in pathogenic potential among respiratory viruses.

These findings are consistent with data showing that RSV is the leading cause of severe respiratory disease in young children^27,28^ and highlight the importance of early detection and confirmatory diagnosis of RSV and influenza through PCR testing for timely identification of patients who may require closer clinical monitoring and prioritized care in hospital settings. Notably, these data were collected during the season immediately preceding the widespread introduction of nirsevimab, a monoclonal antibody with extended half-life targeting the RSV fusion protein, which has shown efficacy in protecting infants against RSV-related hospitalizations and severe RSV infections.^29^ Our data showing increased RSV infection severity underscore the significant implications of the immunization campaigns, which should be prioritized worldwide.

Additionally, the proportion of patients vaccinated against influenza in our cohort was remarkably low. This low vaccination coverage may negatively influence the association of influenza infection with severity outcomes and should be considered in the interpretation of these data. Vaccination provides significant protection and reduces both the incidence and severity of influenza-like illness,^30^ and outcomes may differ in populations with higher coverage, such as US children, who had a 40.8% coverage rate.^31^ Therefore, these findings also underscore the need to enhance strategies targeting both parents and health care professionals with the aim of increasing influenza vaccination uptake, particularly for high-risk children.

Interestingly, in our univariate analysis, HRV/ERV emerged as a protective factor against severe disease. However, this finding represents a sample of all cases observed across the centers and may not be representative of the entire cohort. HRV/ERV is one of the most common pathogens detected in children with acute respiratory infections^32,33^; however, its impact on disease severity has not been thoroughly elucidated. In addition, the molecular PCR assays used in some participating centers detect HRV and ERV as a combined target, preventing the differentiation between the 2 viruses, which have distinct pathogenic profiles.

Notably, in approximately 5% of patients, no etiology was identified through nasal swab testing despite a clinical presentation suggestive of viral respiratory infection. This finding may be due to false-negative test results, delayed collection of respiratory samples, suboptimal sample quality, or the presence of viruses not included in the PCR assay.

In our cohort, age did not emerge as an independent risk factor for severe disease. Moreover, the average severity score and outcomes, such as length of hospital stay and mortality, did not differ significantly among age groups, suggesting that other factors influence disease severity. Data regarding the role of age on infection severity are conflicting. While some studies suggest that etiology, rather than age, determines the infection severity,^12^ other data indicate that age significantly influences viral load, clinical manifestations, and disease severity.^10,14,34^ Interestingly, it is likely that our findings reflect a general trend to hospitalize infants and younger children with a lower clinical threshold, applying a preventive rather than an interventional approach, not rarely prompted by the fears of caregivers. This tendency may lead to a higher number of hospital admissions in infancy and, consequently, a proportion of severe cases comparable with (and not higher than) that observed in older children, who are typically admitted only in the presence of more severe clinical conditions.

Preexisting chronic conditions were already identified in the literature as major contributors to severe disease or death.^11,14,15,16^ In our cohort, children with comorbidities were associated with a significantly higher risk of severe infection and an increased risk of ICU admission. Among them, children with cardiac or pulmonary diseases, congenital malformations, and endocrine or metabolic disorders showed more severe CSS values, suggesting a more careful approach in the initial clinical evaluation of those children. Notably, the increased risk conferred by comorbidities was independent of age.

The impact of comorbidities on the severity of viral respiratory infections has been well studied during the COVID-19 pandemic.^17^ A systematic review conducted in 2021 suggested that children with underlying cardiac disease might be at increased risk of severe COVID-19.^18^ Comorbidities were also associated with a higher rate of hospital admission in children with influenza. Among them, the most common comorbidities were neurological, neurodevelopmental, and gastrointestinal disorders, as well as endocrine and metabolic diseases.^11^ Our findings are also consistent with current bronchiolitis guidelines,^35^ which identify children with comorbidities (particularly prematurity, chronic lung disease, neurological disorders, and immunodeficiency) at increased risk for severe bronchiolitis and emphasize the need for close clinical monitoring and possibly targeted preventive strategies in children with chronic health issues.

In our cohort, the coinfection rate was approximately 40%, slightly higher than those reported in most pediatric studies, in which the simultaneous detection of 2 or more respiratory viruses typically ranged from 10% to 30%.^36,37,38^ However, in studies that employed nucleic acid amplification techniques, coinfection rates exceeding 40% have been reported.^39^ The use of advanced molecular techniques has significantly improved the sensitivity of viral detection in respiratory samples. However, these methods can identify viral genetic material even long after the acute phase of infection has resolved, making it difficult to determine whether a positive test truly reflects an active role by individual agents or a bystander role compared with a direct pathogenic effect by other agents.^40^ It also must be considered that some viruses may exhibit prolonged viral shedding, particularly in immunocompromised individuals, and that certain bacterial coinfections may be related to colonization rather than true infection. Respiratory coinfections were most frequent in children aged 3 to 60 months, compared with both younger and older children. This age pattern is consistent with increased exposure to pathogens through community interactions (eg, family, day care, and school settings) during a time when children encounter many pathogens for the first time in their lives and are particularly susceptible to novel infections.

In our cohort, neither viral-viral nor viral-bacterial coinfections were associated with an increased risk of more severe disease compared with single viral infections. Several studies on respiratory infections in children have reported longer length of hospital stay; an increased risk of hospitalization, ICU admission, and need for mechanical ventilation; and even higher mortality when 2 or more respiratory viruses were detected.^13,38,41^ Conversely, other studies,^42,43^ including reviews and meta-analyses,^44,45^ did not find an association between viral coinfection and such outcomes. This suggests that, in hospitalized children, the presence of multiple pathogens may not necessarily translate into worse clinical outcomes. However, further studies are warranted to explore the potential synergistic effects of coinfections.

### Strengths and Limitations

The strength of our study lies in the multicenter design and the comprehensive data collection encompassing clinical, laboratory, and virological parameters within a well-defined pediatric population. In addition, including multiple viruses is useful for defining clinical implications, reflecting a real-world scenario in which children are hospitalized based on symptoms and medical history, often without a confirmed etiological diagnosis. This perspective is particularly relevant for optimizing health care resource allocation, as it aligns more closely with the initial decision-making process, which is commonly made based on age and comorbidities rather than viral etiology. Furthermore, integrating a prospective clinical surveillance network with real-time data sharing enabled early detection and characterization of infectious threats.

Our study also has some limitations. The study is limited to hospitalized children and therefore reflects moderate-to-severe cases, potentially excluding milder infections managed in outpatient settings. Additionally, as a multicenter study, the retrospective nature of the study and the inclusion of data from different Italian hospitals raise potential concerns about selection bias and the generalizability of the findings. To mitigate this, we implemented a standardized minimum set of investigations for all enrolled patients. Despite previous studies suggesting a pathogenic role of HRV in children younger than 2 years in the presence of high viral loads, viral load data were not available in our cohort, thus we cannot exclude the presence of HRV/ERV carriers and define a real pathogenic role.^32^ This limitation should be considered when interpreting these findings. However, surveillance data from the Centers for Disease Control and Prevention from 2016 to 2021 showed that although RSV and influenza are more frequently detected in inpatient and emergency department settings, HRV/ERV was more frequent in outpatient children, suggesting an association with lower disease severity, consistent with our findings.^33^ Finally, although some 95% CIs in the multivariate analysis are wide, the direction and magnitude of the associations are consistent with findings of previous studies, supporting the robustness of the results.

## Conclusions

In this cohort study, RSV and influenza infections and the presence of chronic comorbidities were associated with severe respiratory disease in hospitalized children. In general, these results should be interpreted within the clinical context, as they may vary by country, health care settings, and socioeconomic status. Similar multicenter studies including both developing and developed countries may be needed to fill this gap and promote generalizability of the results. These results underscore the importance of targeted surveillance and preventive measures, including vaccination and early intervention, particularly for vulnerable pediatric subgroups. The INF-ACT network represents a valuable platform for ongoing monitoring and rapid response to emerging infectious threats in Italy, ultimately contributing to improved pediatric health care outcomes.

## Notes

Supplement 1. **eFigure 1. **Intensive Care Unit Admission, Oxygen Therapy, Length of Hospital Stay, and Clinical Severity Scores by Etiologies **eFigure 2. **Intensive Care Unit Admission, Oxygen Therapy, Length of Hospital Stay, and Clinical Severity Scores by Coinfections **eFigure 3. **Intensive Care Unit Admission, Oxygen Therapy, Length of Hospital Stay, and Clinical Severity Scores by Age **eFigure 4. **Intensive Care Unit Admission, Oxygen Therapy, Length of Hospital Stay, and Clinical Severity Scores by Comorbidities

Supplement 2.
Data Sharing Statement
