# Supplementary material for: Pathogen and Patient Characteristics and the Severity of Viral Respiratory Infections in Children
Source: JAMA Netw Open. 2026 Feb 26;9(2):e260129. doi: 10.1001/jamanetworkopen.2026.0129 (PMC12947030; doi:10.1001/jamanetworkopen.2026.0129)
Supplement: Supplement 1. — eFigure 1. Intensive Care Unit Admission, Oxygen Therapy, Length of Hospital Stay, and Clinical Severity Scores by Etiologies eFigure 2. Intensive Care Unit Admission, Oxygen Therapy, Length of Hospital Stay, and Clinical Severity Scores by Coinfections eFigure 3. Intensive Care Unit Admission, Oxygen Therapy, Length of Hospital Stay, and Clinical Severity Scores by Age eFigure 4. Intensive Care Unit Admission, Oxygen Therapy, Length of Hospital Stay, and Clinical Severity Scores by Comorbidities [file jamanetwopen-e260129-s001.pdf]

Supplementary Online Content

Moracas C, Poeta M, Venturini E, et al. Pathogen and patient characteristics and the severity of viral respiratory infections in children: a comprehensive analysis from the pediatric INF-ACT Network. *JAMA Netw Open*. Published February 26, 2026. doi: 10.1001/jamanetworkopen.2026.0129

eFigure 1. Intensive Care Unit Admission, Oxygen Therapy, Length of Hospital Stay, and Clinical Severity Scores by Etiologies

eFigure 2. Intensive Care Unit Admission, Oxygen Therapy, Length of Hospital Stay, and Clinical Severity Scores by Coinfections

eFigure 3. Intensive Care Unit Admission, Oxygen Therapy, Length of Hospital Stay, and Clinical Severity Scores by Age

eFigure 4. Intensive Care Unit Admission, Oxygen Therapy, Length of Hospital Stay, and Clinical Severity Scores by Comorbidities

This supplemental material has been provided by the authors to give readers additional information about their work.

© 2026 Moracas C et al. JAMA Network Open.

The following figures provide further analyses that supports the interpretation of data included in the main result section, and report additional information about rate of intensive care unit admission, rate of oxygen therapy, mean length of hospital stay and mean Clinical Severity Score values, according to etiology, coinfections, age and comorbidities.

**eFigure 1**	**(a) Rate of Intensive Care Unit admission and oxygen therapy, according to etiology**; [*]P values for ICU admission: P<.001 IV vs other viruses; P=.02 HRV/ERV vs other viruses; [#]P values for oxygen therapy: P=.001 HCoV vs other viruses; P=.02 HAdV vs other viruses; P=.04 HRV/ERV vs other viruses; P<.001 RSV vs other viruses; P=.01 hMPV vs other viruses; P=.02 HBoV vs other viruses. **(b) Mean length of hospital stay and mean Clinical Severity Score values, according to etiology**; [*]P values for length of hospital stay: P=.01 IV vs other viruses; P=.05 HRV/ERV vs other viruses; [#]P values for Clinical Severity Score: P=.002 IV vs other viruses; P=.002 HCoV vs other viruses; P=.008 HAdV vs other viruses; P=.02 HRV/ERV vs other viruses; P<.001 RSV vs other viruses; P=.007 HBoV vs other viruses.<br>**Abbreviations**: ICU, Intensive Care Unit; IV, Influenza; PIV, Parainfluenza virus; HCoV, Human Coronavirus; HAdV, Human Adenovirus; HRV/ERV, Human Rhino/Enterovirus; RSV, Respiratory Syncytial Virus; hMPV, Human Metapneumovirus; HBoV, Human Bocavirus.
**eFigure 2**	**(a) Rate of Intensive Care Unit admission and oxygen therapy, according to coinfections, subdivided into viral respiratory and non-respiratory coinfections, and bacterial coinfections**; [*]P values for ICU admission: P=.005 viral coinfection vs no coinfections, P=.04 respiratory viral coinfection vs no coinfections, P=.03 bacterial coinfection vs no coinfections; [#]P values for oxygen therapy: P=0.003 non-respiratory viral coinfections vs no coinfections. **(b) Mean length of hospital stay and mean Clinical Severity Score values, according to coinfections according to coinfections, subdivided into viral respiratory and non-respiratory coinfections, and bacterial coinfections.**<br>**Abbreviations**: ICU, Intensive Care Unit
**eFigure 3**	**(a) Rate of Intensive Care Unit admission and oxygen therapy, according to age**; [*]P values for ICU admission: P=.04 age >60 months vs other ages; [#]P values for oxygen therapy: P=.05 age 0-3 months vs other ages. **(b) Mean length of hospital stay and mean Clinical Severity Score values, according to age**.<br>**Abbreviations**: ICU, Intensive Care Unit
**eFigure 4**	**(a) Rate of Intensive Care Unit admission and oxygen therapy, according to comorbidities, further subdivided into the following categories: endocrine and inherited metabolic diseases, immune or autoimmune diseases, cardiac or pulmonary diseases, neurological conditions, and congenital malformations**; [*]P values for ICU admission: P<.001 chronic conditions vs no chronic conditions; P=.008 congenital malformations vs no chronic conditions. **(b) Mean length of hospital stay and mean Clinical Severity Score values, according to comorbidities, further subdivided into the following categories: endocrine and inherited metabolic diseases, immune or autoimmune diseases, cardiac or pulmonary diseases, neurological conditions, and congenital malformations**; [*]P values for length of hospital stay: P=.03 chronic conditions vs no chronic conditions; P=.03 endocrine/metabolic diseases vs no chronic conditions; P=.01 congenital malformations vs no chronic conditions. [#]P values for Clinical Severity Score: P=.004 chronic conditions vs no chronic conditions; P=.05 cardiac/pulmonary diseases vs no chronic conditions; P=.02 endocrine/metabolic diseases vs no chronic conditions.<br>**Abbreviations**: ICU, Intensive Care Unit

© 2026 Moracas C et al. JAMA Network Open.

eFigure 1. Intensive Care Unit Admission, Oxygen Therapy, Length of Hospital Stay, and Clinical Severity Scores by Etiologies

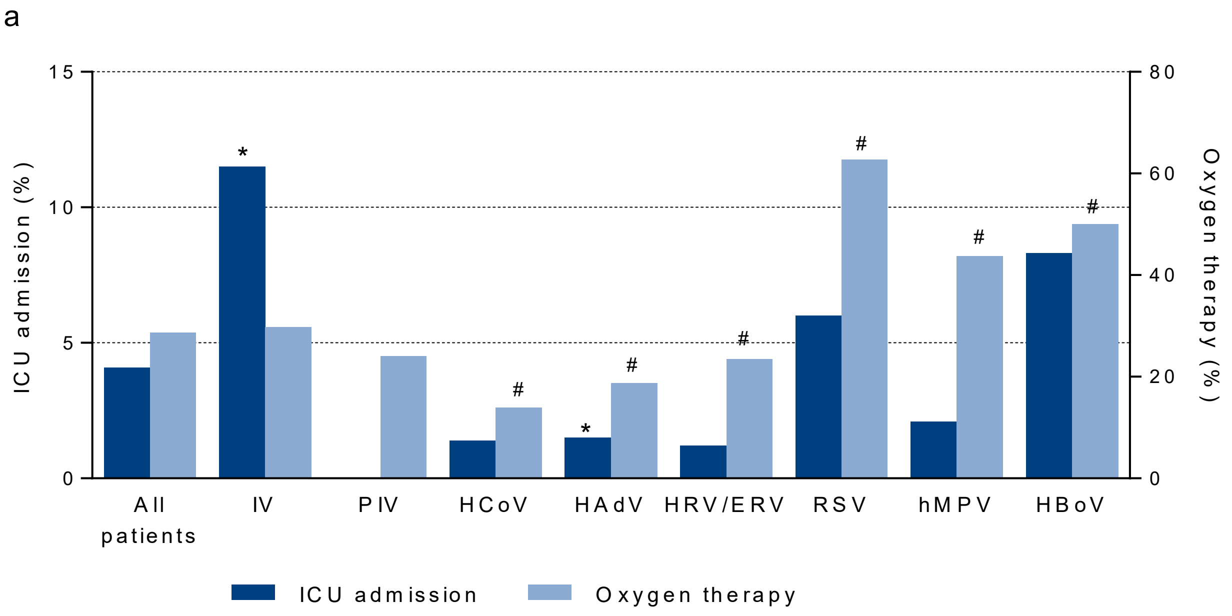

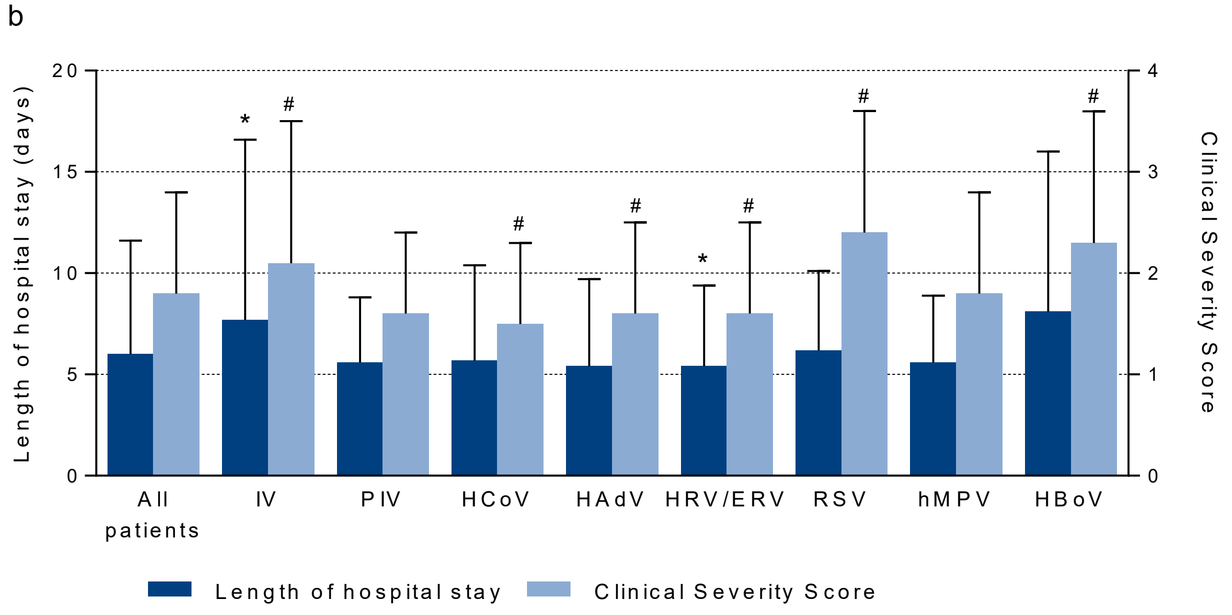

© 2026 Moracas C et al. JAMA Network Open.

eFigure 2. Intensive Care Unit Admission, Oxygen Therapy, Length of Hospital Stay, and Clinical Severity Scores by Coinfections

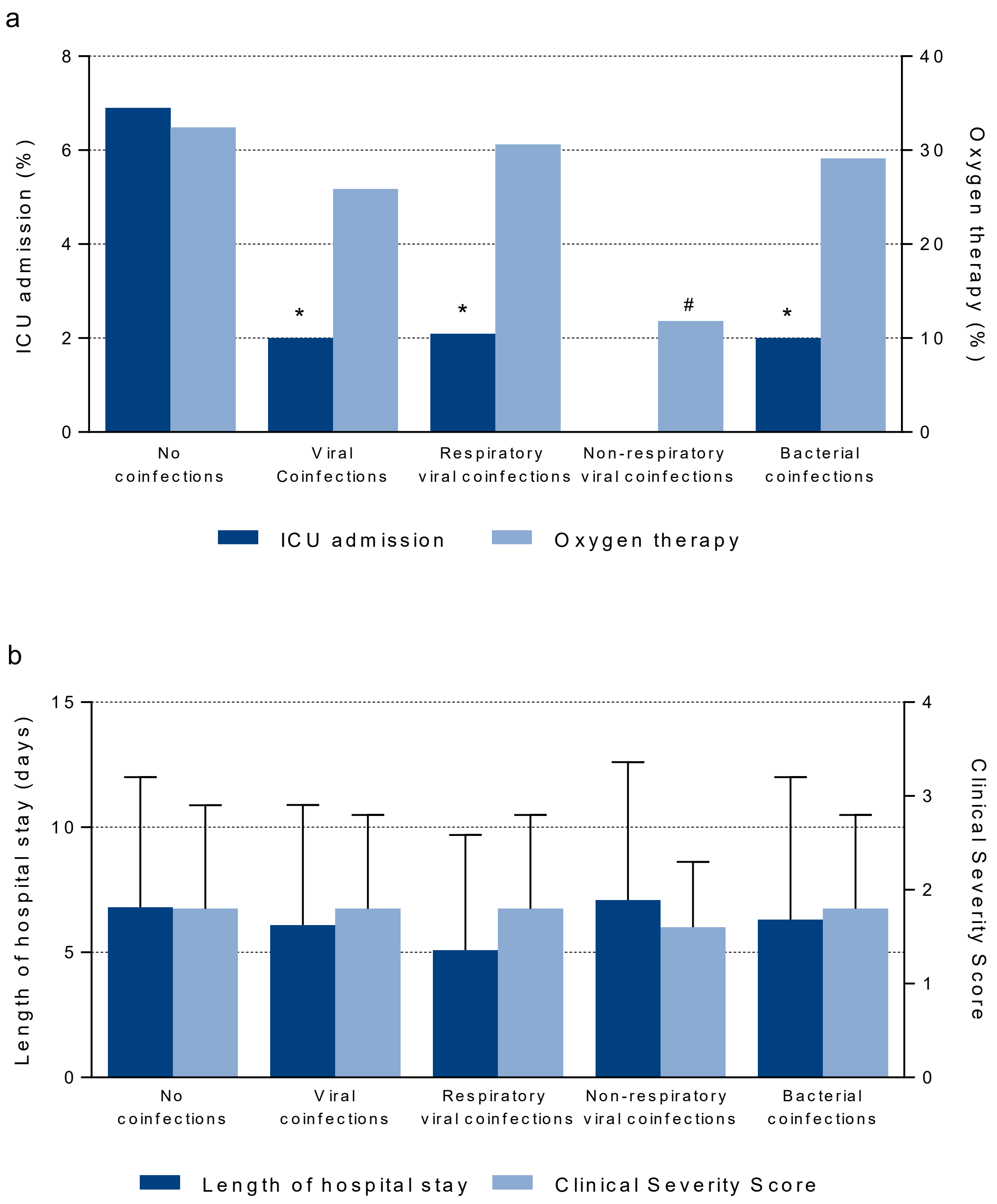

© 2026 Moracas C et al. JAMA Network Open.

eFigure 3. Intensive Care Unit Admission, Oxygen Therapy, Length of Hospital Stay, and Clinical Severity Scores by Age

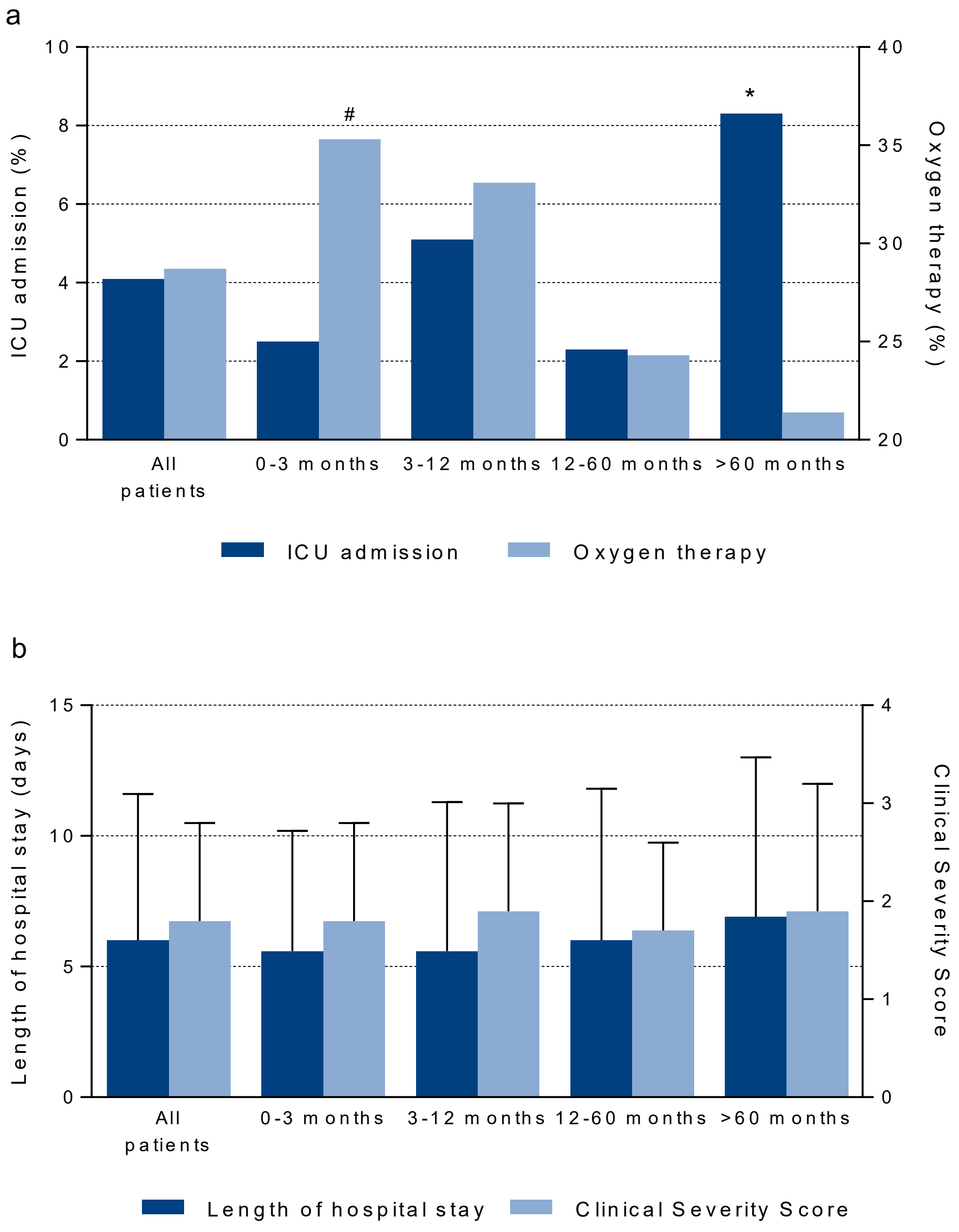

© 2026 Moracas C et al. JAMA Network Open.

eFigure 4. Intensive Care Unit Admission, Oxygen Therapy, Length of Hospital Stay, and Clinical Severity Scores by Comorbidities

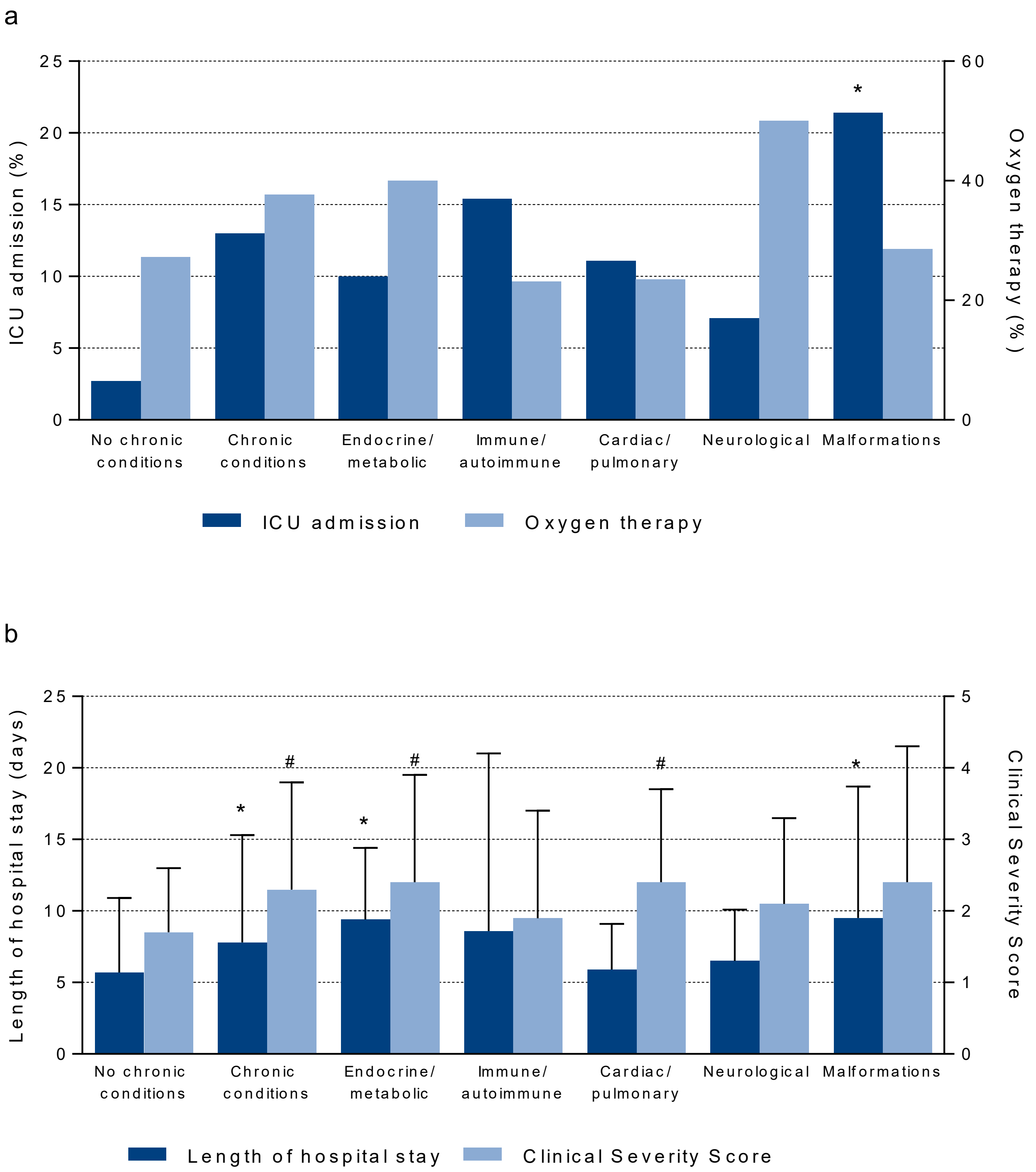

© 2026 Moracas C et al. JAMA Network Open.
