# Supplementary material for: Pathogen and Patient Characteristics and the Severity of Viral Respiratory Infections in Children
Source: JAMA Netw Open. 2026 Feb 26;9(2):e260129. doi: 10.1001/jamanetworkopen.2026.0129 (PMC12947030; doi:10.1001/jamanetworkopen.2026.0129)
Supplement: Supplement 2. — Data Sharing Statement [file jamanetwopen-e260129-s002.pdf]

Data Sharing Statement

Moracas. Pathogen and Patient Characteristics and the Severity of Viral Respiratory Infections in Children. *JAMA Netw Open*. Published February 26, 2026.
doi:10.1001/jamanetworkopen.2026.0129

Data
Data available: Yes
Data types: Deidentified participant data
How to access data: alfguari@unina.it
When available: With publication

Supporting Documents
Document types: Informed consent form
How to access documents: alfguari@unina.it
When available: With publication

Additional Information
Who can access the data: researchers whose proposed use of the data has been approved
Types of analyses: for any purpose
Mechanisms of data availability: with investigator support
